# Long noncoding RNAs and circular RNAs as potential diagnostic biomarkers of inflammatory bowel diseases: a systematic review and meta-analysis

**DOI:** 10.3389/fimmu.2024.1362437

**Published:** 2024-03-08

**Authors:** Melaku Ashagrie Belete, Selamyhun Tadesse, Mihret Tilahun, Alemu Gedefie, Agumas Shibabaw, Zewudu Mulatie, Muluken Amare Wudu, Saba Gebremichael, Habtu Debash, Mihreteab Alebachew, Ermiyas Alemayehu

**Affiliations:** ^1^ Department of Medical Laboratory Sciences, College of Medicine and Health Sciences, Wollo University, Dessie, Ethiopia; ^2^ Department of Medical Laboratory Science, College of Health Sciences, Woldia University, Woldia, Ethiopia; ^3^ Department of Pediatric and Child Health Nursing, School of Nursing and Midwifery, College of Medicine and Health Sciences, Wollo University, Dessie, Ethiopia

**Keywords:** lncRNAs, circRNAs, diagnostic biomarkers, inflammatory bowel disease, ulcerative colitis, Crohn’s disease, meta-analysis

## Abstract

**Introduction:**

Inflammatory bowel disease (IBD) poses a growing global burden, necessitating the discovery of reliable biomarkers for early diagnosis. The clinical significance of dysregulated expression of long noncoding RNAs (lncRNAs) and circular RNAs (circRNAs) in diagnosing IBD has not been well established. Thus, our study aimed to investigate the diagnostic value of lncRNAs and circRNAs for IBD based on currently available studies.

**Methods:**

A comprehensive search was carried out in diverse electronic databases, such as PubMed, Embase, Scopus, Science Direct and Wiley Online Library to retrieve articles published until October 30, 2023. Stata 17.0 software was employed to determine pooled sensitivity, specificity, positive likelihood ratio (PLR), negative likelihood ratio (NLR), diagnostic ratio (DOR), and area under the curve (AUC). Heterogeneity, subgroup analysis, and meta-regression were explored, and publication bias was assessed using Deeks’ funnel plot. Fagan’s nomogram and likelihood ratio scattergram were employed to evaluate the clinical validity.

**Result:**

A total of 11 articles encompassing 21 studies which involved 1239 IBD patients and 985 healthy controls were investigated. The findings revealed lncRNAs exhibit high level of pooled sensitivity 0.94 (95% CI: 0.87-0.97) and specificity 0.99 (95% CI: 0.89-1.00), along with PLR, NLR, DOR, and AUC values of 64.25 (95% CI: 7.39-558.66), 0.06 (95% CI: 0.03-0.13), 1055.25 (95% CI: 70.61-15770.77), and 0.99 (95% CI: 0.97-0.99), respectively. Conversely, CircRNAs showed moderate accuracy in IBD diagnosis, with sensitivity of 0.68 (95% CI: 0.61-0.73), specificity of 0.73 (95% CI: 0.65-0.79), PLR of 2.47 (95% CI: 1.94-3.16), NLR of 0.45 (95% CI: 0.38-0.53), DOR of 5.54 (95% CI: 3.88-7.93), and AUC value of 0.75 (95% CI: 0.71-0.79). Moreover, findings from subgroup analysis depicted heightened diagnostic efficacy when employing lncRNA H19 and a large sample size (≥100), with notable efficacy in diagnosing both ulcerative colitis (UC) and Crohn’s disease (CD).

**Conclusion:**

LncRNAs exhibit high diagnostic accuracy in distinguishing patients with IBD from healthy controls signifying their possible use as potential biomarkers, while circRNAs showed moderate diagnostic accuracy. Nevertheless, to validate our findings and confirm the clinical utility of lncRNAs and circRNAs in IBD diagnosis, a large pool of prospective and multi-center studies should be undertaken.

**Systematic review registration:**

https://www.crd.york.ac.uk/PROSPERO, identifier CRD42023491840.

## Introduction

Inflammatory bowel disease is primarily composed of ulcerative colitis (UC), which is confined to the mucosa of the colon, and Crohn’s disease (CD), which can impact any part of the gastrointestinal tract (GIT) from the mouth to the anus. These are two phenotypes within the GIT that share a persistent state of inflammation but vary in symptoms, the location of the disease, and histopathological features (1–3). The pathogenesis of IBD remains unclear, with various factors potentially interacting to contribute to its development. These factors include genetic predisposition, microbial infection, and environmental influences (4). Genetic susceptibility, environmental factors (such as diet, smoking, and microbial exposure), the presence of pathogenic factors such as abnormal gut microbiota, and dysregulated immune responses including cytokines (such as TNFα) and immune cells with exaggerated inflammatory responses contribute to chronic inflammation in the GIT with repeated cycles of relapse and remission, leading to the characteristic symptoms of IBD (5). Currently, IBD treatments include anti-inflammatory drugs (amino salicylates and corticosteroids), immunomodulators (thiopurines, methotrexate, and newer biologic agents (e.g., anti-TNF drugs like infliximab), biological therapies (monoclonal antibodies targeting specific inflammatory pathways such as anti-integrins and interleukins), and surgery (6). However, these are not entirely curative treatments for IBD. While these treatments can effectively induce and maintain remission, challenges exist, including variable response rates, side effects, and the need for personalized treatment approaches. Additionally, the chronic nature of IBD necessitates long-term management strategies (7, 8).

Between 1990 and 2017, there was a substantial increase in the prevalence of IBD, rising from 3.7 million to over 6.8 million. This marked an 85.1% global rise in IBD cases during that timeframe. Furthermore, across these years, the prevalence rate consistently demonstrated a significant predominance in females over males, with females constituting 57% and males making up 43% (9). IBD exerts profound effects on patients, impacting their quality of life, mental health, work productivity, and the utilization of healthcare resources (10, 11). Additionally, the number of deaths related to IBD increased by 67.0% from 1990 to 2017, rising from 23,000 to 38,000 (9).

The complexity and variability of symptoms associated with IBD make it challenging to establish a singular “gold standard” test for diagnosis, severity assessment, or treatment response evaluation. Physicians adopt a comprehensive approach, considering clinical symptoms, laboratory indices, radiological investigations, endoscopy, and histological examination of tissue specimens to thoroughly assess disease activity and formulate appropriate treatment strategies (12, 13). Extensive studies over the past decades have focused on laboratory indices for IBD, leading to the integration of specific biomarkers into clinical practice (14). Recent progress in biomarker discovery for IBD emphasizes the integration of cutting-edge proteomic techniques through innovations such as liquid chromatography-mass spectrometry (LC-MS), electrospray ionization (ESI) and tandem mass spectrometry (MS) that allow distinct protein signatures associated with IBD subtypes, employing high-throughput proteomic analyses (15, 16). While the integration of proteomic data with genomics and transcriptomics hold promises for accurate diagnosis and personalized treatment, challenges such as standardization, reproducibility, and validation persist (17). Currently, blood and fecal biomarkers such as anti-neutrophil cytoplasm antibody, anti-laminaribioside carbohydrate antibody, C-reactive protein, fecal calprotectin, and anti-Saccharomyces cerevisiae antibody are employed in the management of IBD. However, their utility is limited in the initial diagnosis of IBD due to their low specificity or sensitivity (18, 19). Nevertheless, despite these advancements, there is still no ideal biomarker possessing all the necessary qualities for the precise diagnosis of IBD, differentiation between IBD subtypes, or effective monitoring of disease activity (20). Therefore, discovering ideal diagnostic biomarkers for IBD is crucial. Optimal biomarkers are expected to be non-invasive, sensitive, disease-specific, easy to perform, and cost-effective (14).

Non-coding RNAs (ncRNAs), including long non-coding RNAs (lncRNAs), microRNAs (miRNAs), and circular RNAs (circRNAs), constitute a diverse set of transcripts that are not translated into proteins. Since their discovery, ncRNAs have emerged as significant regulators of various biological functions across different cell types and tissues, and their dysregulation has been associated with disease (21). Noncoding RNAs, exert regulatory control through diverse mechanisms. LncRNAs influence gene expression at transcriptional and post-transcriptional levels by interacting with chromatin and RNA binding proteins, while miRNAs primarily act post-transcriptionally by binding to the 3’ untranslated region (UTR) of target mRNAs, and hinder translation initiation by preventing ribosome binding to target mRNAs. CircRNAs function as competitive endogenous RNAs (ceRNAs) by acting as miRNA sponges. Collectively, these ncRNAs form intricate regulatory networks, and employ diverse mechanisms to regulate biological functions, from influencing chromatin dynamics to fine-tuning gene expression at the post-transcriptional level, impacting cellular processes crucial for understanding diseases like IBD and exploring diagnostic potential (22). Recent literature has uncovered evolving associations between IBD and noncoding RNAs, recognizing them as pivotal regulators of gene expression at both transcriptional and post-transcriptional levels (23). While certain noncoding RNAs play a protective role by preserving gut microbiota homeostasis (24) and controlling intestinal inflammation, the majority are implicated in the pathogenesis of IBD through disruptions in autophagy, the intestinal barrier, and immune homeostasis (25).

Multiple studies have emphasized the notable contribution of dysregulated lncRNAs and circRNAs in diagnosing IBD, indicating that both types of RNAs serve as effective advanced diagnostic biomarkers with increased sensitivity and specificity for IBD diagnosis (26–31). Besides, there are reports highlighting distinct patterns in the relative expression levels of lncRNAs and circRNAs among CD and UC patients (32–35). More importantly, the expression profiling of both parameters in individuals with IBD has produced inconsistent results. Given these varied insights, a comprehensive analysis is imperative to evaluate the applicability of these biomarkers as diagnostic tools for IBD. Therefore, this systematic review and meta-analysis aimed to assess the overall diagnostic accuracy of lncRNAs and circRNAs for IBD.

## Methods

### Study protocol

This study adheres to the commendations specified in the Preferred Reporting Items for Systematic Reviews and Meta-analysis (PRISMA) guideline (36) as indicated in **Supplementary File 1**. The study protocol was officially registered in the Prospective Register of Systematic Reviews (PROSPERO) with the registration identification number of CRD42023491840.

### Literature search strategy and data sources

A systematic and thorough search of the existing literature on the diagnostic efficacy of lncRNAs and circRNAs for IBD was conducted by two independent researchers (MAB and EA) using diverse electronic bibliographic databases, such as PubMed, Embase, Scopus, Science Direct and Wiley Online Library. Furthermore, a manual search on Google was executed to uncover any pertinent studies possibly overlooked in the electronic database searches, by scrutinizing the bibliographies of the identified studies. The conclusive search took place on October 30, 2023. The search strategy incorporated Medical Subject Heading (MeSH) terms and keywords, such as “long noncoding RNA”, “long non-coding RNA”, “long non coding RNA”, “long ncRNA”, “long ncRNAs”, “ncRNAs, long”, “lncRNA”, “lnc RNA”, “ncRNA”, “RNA, long noncoding”, “noncoding RNA, long”, “circular RNA”, “circular RNAs”, “RNA, circular”, “circRNA”, “circRNAs”, “diagnos*”, “inflammatory bowel disease*”, “Crohn*”, “ulcerative colitis”. Boolean operators (“OR” and “AND”) were used as necessary in the advanced search databases. The detailed search strategy is available in the **Supplementary File 2**.

### Eligibility criteria

This review focused on specific categories of research, specifically observational studies (including cross-sectional, case-control and cohort studies) published until October 30, 2023, which investigated the potential of lncRNA and circRNA as a diagnostic marker for differentiating between IBD patients and healthy individuals. Furthermore, the selected studies were required to provide crucial information such as sensitivity, specificity, and sample sizes, facilitating the computation of key diagnostic metrics like true positives (TP), false positives (FP), false negatives (FN), and true negatives (TN). Conversely, the review excluded various types of articles, such as review articles, case reports, narrative reviews, conference abstracts, editorials, commentaries, letters to the editor, and author replies. Additionally, studies lacking human subjects or essential data for calculating TP, FP, TN, and FN were excluded. These defined inclusion and exclusion criteria were applied to guide the study selection process for the meta-analysis.

### Study selection and data extraction

The literature search results were imported into EndNote 20 software (Clarivate Analytics USA) and duplicate were removed. Following this, a comprehensive screening process was conducted for each chosen article involving the assessment of the title, abstract, and full text by two independent reviewers (MAB and EA), adhering to pre-established eligibility criteria. In instances where discrepancies or disagreements arose between the two reviewers, a discussion ensued, and a third reviewer (AG) was brought in as necessary to make final determinations regarding the inclusion of articles in the review.

Vital data from the eligible studies were extracted into an Excel spreadsheet using a preconceived data abstraction form. Three researchers (MAB, EA and MT) extracted data including first author name, publication year, study country, extracted lncRNA or circRNA type, lncRNA or circRNA expression pattern, specimen type, internal reference control, sample sizes, sex and age for both IBD patients and healthy individuals, diagnostic methods, and cut-off values. Moreover, diagnostic parameters like sensitivity, specificity, and the area under the curve (AUC) were extracted. The three reviewers meticulously reviewed and verified their extraction results. Any disparities between the data extractors were resolved through discussion and consensus, involving a third reviewer (ST). This process ensured the integrity of the collected data.

### Quality assessment

Two independent evaluators (ZM and ST) carried out a thorough assessment of the methodological and substantive quality of eligible studies using the modified Quality Assessment of Diagnostic Accuracy Studies-2 (QUADAS-2) tool, implemented with the support of Review Manager (RevMan) 5.3 software (37). The QUADAS-2 tool consists of four domains: patient selection, index test, reference standard, and flow and timing. The process involved appraising the clinical relevance of selected patients, the performance of the index test, and the adequacy of the reference standard. The overall risk of bias for the comparison can then be assessed by considering the risk of bias for each domain. The resulting risk of bias was then categorized as low (L), high (H), or unclear (U) based on the outcomes of this evaluation.

### Statistical analysis

Stata version 17.0 software (Stata Corp., College Station, TX) was used for analyzing extracted data. To gauge and assess heterogeneity among the studies, the Cochrane Q test and I^2^ statistics were employed. Significant heterogeneity was identified when the I^2^ test statistic values exceeded 50%, and the p-value was less than 0.05. The extracted data from each eligible study were transformed into diagnostic parameters, encompassing true positives (TP), false positives (FP), false negatives (FN), and true negatives (TN). These parameters were utilized to compute pooled sensitivity, specificity, positive likelihood ratio (PLR), negative likelihood ratio (NLR), diagnostic odds ratio (DOR), and area under the curve (AUC) through a random-effects model. The overall diagnostic accuracy of lncRNA and circRNA in diagnosing IBD was assessed using AUC and DOR from the summary receiver characteristic curve (SROC). The presence of a threshold effect was established by analyzing the Spearman correlation coefficient and visually inspecting the SROC curve. A p-value of less than 0.05 derived from the Spearman correlation coefficient, combined with the absence of the characteristic “shoulder-arm” shape in the SROC curve, indicated the presence of a threshold effect. Subgroup analyses and meta-regression analyses were conducted to explore the primary sources of heterogeneity. The presence of publication bias was assessed using Deeks’ funnel plot asymmetry, where a p-value greater than 0.10 indicated the absence of publication bias. Furthermore, the clinical utility of lncRNAs and circRNAs in distinguishing IBD patients from healthy individuals was assessed deploying the Fagan plot and likelihood ratio scattergram.

## Results

### Selection of studies

**Figure 1:**
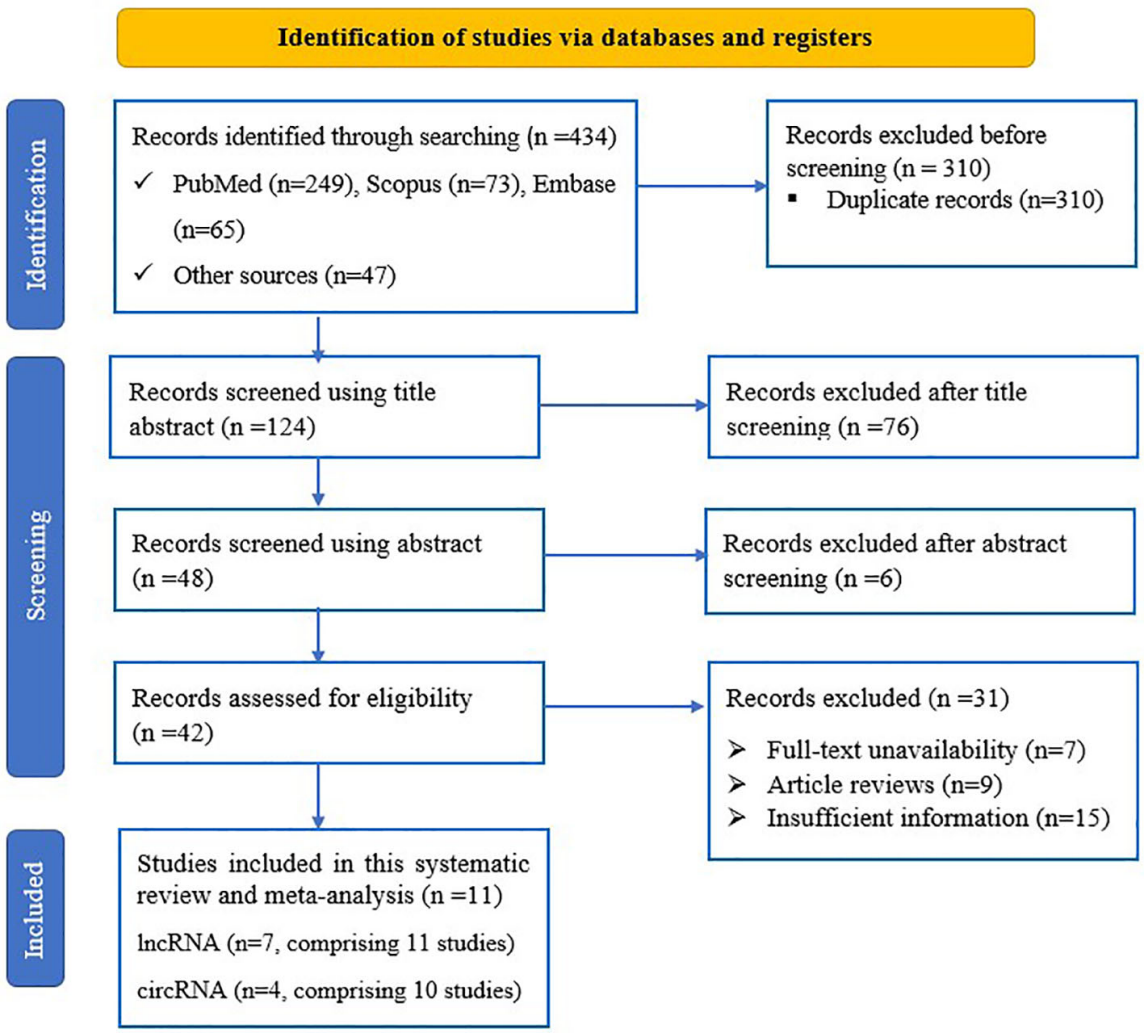
PRISMA flow diagram of eligible study selection process for the systematic review and meta-analysis.

Upon conducting the primary search of the databases and other sources, a total of 434 records were retrieved. Following the removal of duplicates, the remaining 124 articles were screened based on review of title and abstract, and 82 were removed. After conducting an in-depth review, a total of 42 articles were deemed suitable for full-text analysis, and were thoroughly evaluated against the eligibility criteria, resulting in the exclusion of 31 studies for various reasons. Ultimately, after this rigorous process, 21 studies from 11 sources (31, 32, 38–46) were found to be potentially eligible for inclusion in the meta-analysis (**Figure 1**).

### Characteristics of included studies

This systematic review and meta-analysis included data on lncRNAs from a total of 11 studies, involving 531 individuals diagnosed with IBD and 461 healthy controls whereas 10 studies were included regarding circRNAs encompassing a total of 708 IBD patients and 524 healthy controls. Majority of the included studies were from China, followed by Egypt, and used serum sample for lncRNA and circRNA detection. All studies deployed quantitative real-time PCR (qRT-PCR) for assessing lncRNA and circRNA expression. Concerning lncRNA profiling, 9 studies focused on individual lncRNA, while 2 studies explored combined lncRNAs. Among the findings, eight studies reported an upregulation in lncRNA expression, whereas three studies reported a downregulation. Among ten lncRNA, three lncRNAs (lncRNA H19, lncRNA THRIL, lncRNA PVT1) were reported to be upregulated. One lncRNA (lncRNA ANRIL) was downregulated in serum specimens of IBD patients (**Table 1A**). On the other hand, all ten studies focused on individual circRNA, reported upregulation of circRNA expression, and used β-Actin as an internal reference (**Table 1B**).

**Table 1A:** Characteristics of the included studies for long noncoding RNAs.

Authors	Year	Country	Specimen	Method	Reference	Participants						lncRNA	Expression	Cut-off	Sen (%)	Spec (%)	AUC
						Case	No (male/female)	Age (mean± SD)	Control	No (male/female)	Age (mean SD)						
Shaker et al. (38)	2023	Egypt	Serum	qRT-PCR	GAPDH	UC	35 (21/14)	32.06 ± 1.9	HC	30 (15/15)	32.07 ± 0.8	LncRNA H19	Up	3.53	94.3	90.0	0.944
Shaker, et al. (38)	2023	Egypt	Serum	qRT-PCR	GAPDH	CD	32 (21/11)	33.84 ± 2.1	HC	30 (15/15)	32.07 ± 0.8	LncRNA H19	Up	2.04	87.5	88.5	0.875
Elamir, et al. (39)	2022	Egypt	Serum	qRT-PCR	GADPH	UC	70 (42/28)	32.10 ± 3.7	HC	70 (45/25)	30 ± 2.5	LncRNA THRIL	Up	4.62	100	100	1.000
Elamir, et al. (39)	2022	Egypt	Serum	qRT-PCR	GADPH	CD	70 (44/26)	33.20 ± 1.7	HC	70 (45/25)	30 ± 2.5	LncRNA THRIL	Up	1.57	100	100	1.000
Khalil, et al. (40)	2023	Egypt	Serum	qRT-PCR	NA	UC	34 (20/14)	31.79 ± 11.2	HC	40 (25/15)	27.00 ± 7.3	LncRNA H19	Up	NA	94.12	100	0.944
Khalil, et al. (40)	2023	Egypt	Serum	qRT-PCR	NA	CD	36 (21/15)	29.59 ± 8.9	HC	40 (25/15)	27.00 ± 7.3	lncRNA H19	Up	NA	88.24	96.67	0.909
Mirza, et al. (41)	2015	Denmark	Biopsy	qRT-PCR	GAPDH	UC	20 (7/13)	44 ± 39.9	HC	12 (4/8)	49.66 ± 49.5	MMP12, RP11-731 F5.2, AC007182.6, DPP10-AS1, CDKN2B-AS1, and AL928742.12	Down	NA	86.7	100	
Mirza, et al. (41)	2015	Denmark	Biopsy	qRT-PCR	GAPDH	CD	13 (7/6)	36.33 ± 33.2	HC	12 (4/8)	49.66 ± 49.5	MMP12, RP11-731 F5.2, AC007182.6, DPP10-AS1, CDKN2B-AS1, and AL928742.12	Down	NA	100	100	
Sobhy, et al. (42)	2023	Egypt	Serum	qRT-PCR	GAPDH	UC	60 (36/24)	32.10 ± 1.99	HC	30 (16/14)	31.06 ± 1.04	LncH19	Up	2.659	91.7	99.3	0.917
Ge, et al. (43)	2019	China	Intestinal mucosa	qRT-PCR	GAPDH	UC	101 (38/63)	34.1 ± 8.6	HC	67 (32/35)	35.6 ± 9.6	LncRNA ANRIL	Down	NA	86.1	64.2	0.803
Ayoup, et al. (44)	2021	Egypt	Serum	qRT-PCR	GADPH	UC	60 (28/32)	38.8 ± 5.9	HC	60 (26/34)	39.6 ± 5.5	LncRNA PVT1	Up	1.06	73.3	83.3	0.784

HC, healthy control; CD, Crohn’s disease; UC, ulcerative colitis; GAPDH, Glyceraldehyde 3-Phosphate Dehydrogenase; NA, not available; NGS, next generation sequence; qRT-PCR, quantitative real-time polymerase chain reaction; Sen, sensitivity; Spec, specificity; AUC, area under curve.

**Table 1B:** Characteristics of the included studies for circular RNAs.

Authors	Year	Country	Specimen	Method	Reference	Participants						circRNA	Expression	Cut-off	Sen (%)	Spec (%)	AUC
						Case	No (male/female)	Age (mean± SD)	Control	No (male/female)	Age (mean SD)						
Ye et al. (31)	2019	China	PBMCs	qRT−PCR	β-Actin	CD	90 (48/42)	39.9± 11.8	HC	80 (46/34)	37.6± 9.3	circRNA_103516	Up	1.412	66.67	78.75	0.790
Ye et al. (31)	2019	China	PBMCs	qRT−PCR	β-Actin	UC	90 (38/52)	41.7± 12.4	HC	80 (46/34)	37.6± 9.3	circRNA_103516	Up	1.151	66.67	62.50	0.687
Yin et al. (32)	2019	China	PBMCs	qRT−PCR	β-Actin	CD	87 (52/35)	40.9± 35.4	HC	55 (33/22)	38.9± 33.5	circRNA_092520	Up	0.00076	52.63	73.08	0.66
Yin et al. (32)	2019	China	PBMCs	qRT−PCR	β-Actin	CD	87 (52/35)	40.9± 35.4	HC	55 (33/22)	38.9± 33.5	circRNA_102610	Up	0.00154	60.53	78.85	0.78
Yin et al. (32)	2019	China	PBMCs	qRT−PCR	β-Actin	CD	87 (52/35)	40.9± 35.4	HC	55 (33/22)	38.9± 33.5	circRNA_004662	Up	0.00035	65.52	90.91	0.85
Yin et al. (32)	2019	China	PBMCs	qRT−PCR	β-Actin	CD	87 (52/35)	40.9± 35.4	HC	55 (33/22)	38.9± 33.5	circRNA_103124	Up	0.00481	71.05	65.38	0.74
Ye et al. (46)	2021	China	PBMCs	qRT−PCR	β-Actin	CD	60 (37/23)	35.9± 10.3	HC	40 (21/19)	38.2± 10.8	circRNA_103765	Up	NA	53.33	77.50	0.701
Ye et al. (46)	2021	China	PBMCs	qRT−PCR	β-Actin	UC	60 (33/27)	40.3± 11.6	HC	40 (21/19)	38.2± 10.8	circRNA_103765	Up	NA	80.00	45.00	0.652
Hu et al. (45)	2021	China	Colon tissue	qRT-PCR	β-Actin	CD	30 (17/13)	36.3± 12.5	HC	32 (21/11)	42.6± 7.0	hsa_circ_0001666	Up	NA	83.30	78.80	0.858
Hu et al. (45)	2021	China	Colon tissue	qRT-PCR	β-Actin	CD	30 (17/13)	36.3± 12.5	HC	32 (21/11)	42.6± 7.0	hsa_circ_0062142	Up	NA	83.30	67.30	0.803

HC, healthy control; CD, Crohn’s disease; UC, ulcerative colitis; PBMCs, Peripheral blood mononuclear cells; β-Actin, beta actin gene; NA, not available; NGS, next generation sequence; qRT-PCR, quantitative real-time polymerase chain reaction; Sen: sensitivity; Spec, specificity; AUC, area under curve.

### Quality assessment of studies included in meta-analysis

**Figure 2:**
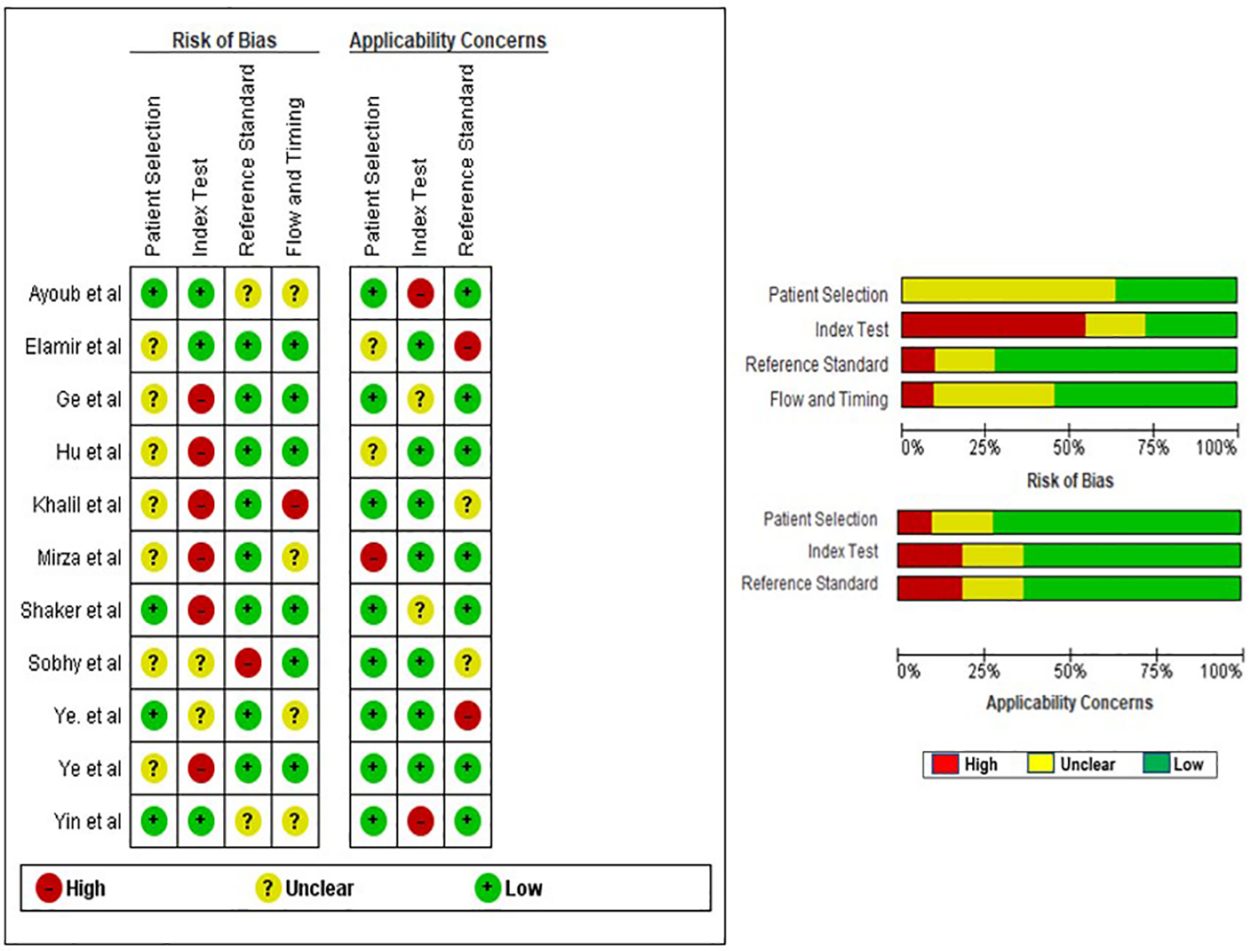
Risk of bias assessment of eligible studies using QUADAS-2.

The evaluation of the quality of the eligible studies was carried out through the utilization of the QUADAS-2 tool. Due to the crucial role of patient selection in ensuring experimental integrity, the data incorporated into this meta-analysis predominantly came from well-validated groups. In general, the studies included demonstrated satisfactory and qualifying methodological standards. A comprehensive breakdown of the criteria used in the quality assessment is clearly shown in **Figure 2**.

### Overall diagnostic accuracy of lnc and circular RNA in diagnosing IBD

The presence of a threshold effect of heterogeneity was evaluated using both the Spearman correlation coefficient and the SROC curve. The findings from the Spearman correlation coefficient depicting a rho value of -0.33 and a p-value of 0.34, along with the absence of the characteristic “shoulder-arm” shape in the SROC curve, indicated that there is no evidence of a threshold effect of heterogeneity. Moreover, the I^2^ values for sensitivity, specificity, PLR, NLR, and DOR for lncRNA markers were 91.69%, 96.85%, 93.47%, 92.78%, and 100%, respectively. Conversely, the I^2^ values for sensitivity, specificity, PLR, NLR, and DOR for circRNA markers were 66.11%, 73.11%, 39.82%, 47.22%, and 98.44%, respectively. In both cases, given that the I^2^ results surpass 50% and the p-values for all parameters are below 0.001, it strongly indicates the existence of significant non-threshold effect heterogeneity in this study. Consequently, a random-effects model was utilized for the meta-analysis.

**Figure 3:**
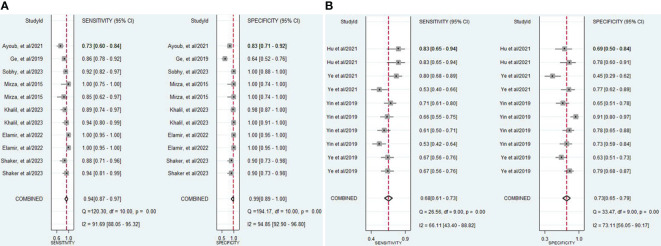
**(A)** Forest plot of pooled sensitivity and specificity of lncRNAs in diagnosing of IBD. **(B)** Forest plot of pooled sensitivity and specificity of circRNAs in diagnosing of IBD.

**Figure 4:**
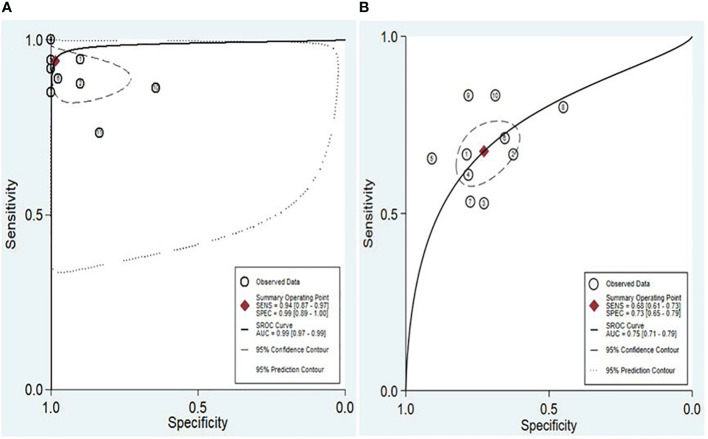
SROC and the 95% confidence contour and 95% prediction contour for lncRNAs **(A)** and circRNAs **(B)**.

The findings revealed that both lncRNAs and circRNAs demonstrated strong diagnostic potential for detecting IBD. The combined sensitivity and specificity for lncRNA markers were 0.94 (95% CI: 0.87-0.97) and 0.99 (95% CI: 0.89-1.00), respectively (**Figure 3A**). Similarly, the combined sensitivity and specificity for circRNA markers were 0.68 (95% CI: 0.61-0.73) and 0.73 (95% CI: 0.65-0.79), respectively (**Figure 3B**). Additionally, the pooled PLR and NLR were 64.25 (95% CI: 7.39-558.66) and 0.06 (95% CI: 0.03-0.13), respectively for lncRNA, and 2.47 (95% CI: 1.94-3.16) and 0.45 (95% CI: 0.38-0.53), respectively for circRNA. Furthermore, the DOR for lncRNA and circRNA markers were 1055.25 (95% CI: 70.61-15770.77) and 5.54 (95% CI: 3.88-7.93). In assessing diagnostic accuracy of lncRNA and circRNA, the SROC curve was generated, resulting in an AUC of 0.99 (95% CI: 0.97-0.99) and 0.75 (95% CI: 0.71-0.79), respectively (**Figure 4**). These results indicate that both lncRNAs and circRNAs exhibit high diagnostic accuracy in identifying IBD, as an AUC greater than 0.7 is indicative of a strong predictive capability.

### Clinical applicability of lncRNA and circRNA for diagnosing IBD

**Figure 5:**
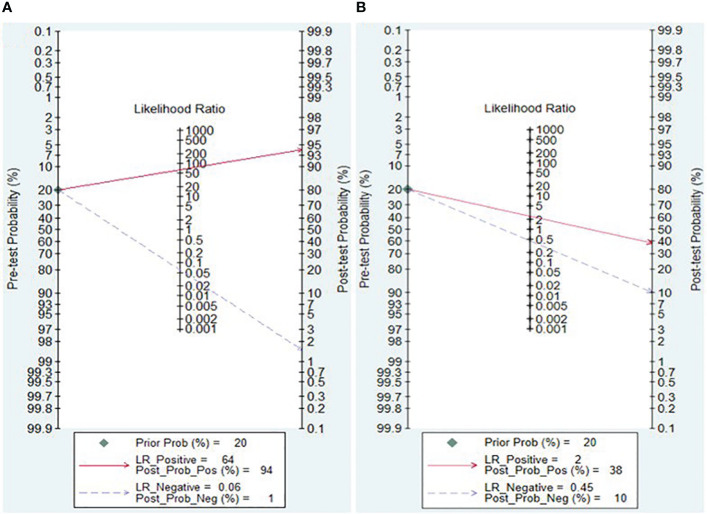
Fagan nomogram and likelihood ratio scattergram for lncRNAs **(A)** and circRNAs **(B)**.

**Figure 6:**
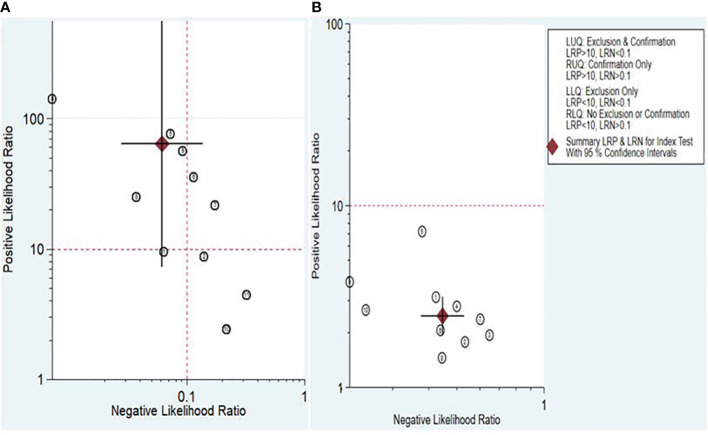
Scattergram assessing the clinical applicability of lncRNAs **(A)** and circRNAs **(B)** for diagnosing IBD.

The Fagan nomogram and likelihood ratio scattergram were employed to evaluate the clinical value of lncRNAs and circRNAs in diagnosing IBD. The Fagan’s nomogram showed promising outcomes, revealing post-test probabilities with PLR and NLR values of 0.94 and 0.01 for lncRNAs and 0.38 and 0.1 for circRNA, respectively, under a pre-test probability set at 20% (**Figures 5A, B**). Furthermore, a scattergram depicting the likelihood ratios (PLR and NLR) was generated to assess the clinical applicability of lncRNAs and circRNA in IBD diagnosis. The results indicated that studies conducted by Khalil et al. (lncRNA H19), Mirza et al. (MMP12, RP11-731 F5.2, AC007182.6, DPP10-AS1, CDKN2B-AS1, and AL928742.12), and Sobby et al. (lncRNA H19) laid over on the left upper quadrant (PLR > 10 and an NLR < 0.1), indicating that the markers could be used for both exclusion and confirmation of IBD (**Figure 6A**). On the other hand, all eligible studies for circRNAs were positioned in the right lower quadrant (PLR < 10 and an NLR < 1), indicating that these markers are not suitable for confirming or excluding IBD (**Figure 6B**).

### Subgroup analysis and meta-regression

Due to the presence of substantial heterogeneity (I^2^ > 50% and P < 0.05) across all diagnostic performance parameters, such as sensitivity, specificity, PLR, NLR, DOR, and AUC, meta-regression and subgroup analyses were carried out. The purpose of these analyses was to delve into the sources of heterogeneity among the studies, examining various study characteristics. These characteristics include country, biological specimen, regulation pattern, lncRNA profiling, sample size, internal reference, cut-off value establishment, and the classification of IBD patients.

In the subgroup analysis, lncRNA H9 showed a relatively lower diagnostic performance for IBD compared to other lncRNAs. Notably, lncRNA H9 demonstrated lower sensitivity (0.91, 95% CI: 0.86-0.95), specificity (0.97, 95% CI: 0.89-0.99), PLR (28.1, 95% CI: 7.9-100.3), NLR (0.09, 95% CI: 0.06-0.14), DOR (314, 95% CI: 75-1309), and AUC (0.93, 95% CI: 0.90-0.95) compared to other lncRNAs (sensitivity: 0.98, 95% CI: 0.77-1.00; specificity: 1.00, 95% CI: 0.29-1.00; PLR: 291, 95% CI: 0.4-2079; NLR: 0.02, 95% CI: 0.01-0.28; DOR: 132, 95% CI: 4-439; AUC: 1.00, 95% CI: 0.99-1.00).

In terms of sample size, lncRNAs exhibited the highest overall diagnostic accuracy when the sample size was >100, with a sensitivity 0.99 (95% CI: 0.46-1.00), specificity 0.99 (95% CI: 0.33-1.00), PLR 104.1 (95% CI: 0.5-219), NLR 0.01 (95% CI: 0.01-1.13), DOR 989 (955 CI: 1-7661), and AUC of 1.00 (95% CI: 0.99-1.00), demonstrating superior diagnostic performance compared to instances where the sample size was ≤100. Furthermore, lncRNAs demonstrated notable efficacy in the diagnosis of both UC and CD with minimal differences in parameters. In case of the CD, the diagnostic parameters were as follows: sensitivity of 0.97 (95% CI: 0.76-1.00), specificity of 0.99 (95% CI:0.70-1.00), PLR of 123.8 (95% CI: 2.8-857), NLR of 0.03 (95% CI: 0.01-0.30), DOR of 476 (95% CI: 15-1548), and an AUC of 1.00 (95% CI: 0.99-1.00) (**Table 2A**). On the other hand, circRNAs exhibited moderate overall diagnostic accuracy when the sample size >100 with sensitivity 0.75 (95% CI: 0.61-0.85), Specificity 0.68 (95% CI: 0.53-0.80), PLR 2.3 (95% CI: 1.6-3.4), NLR 0.37 (95% CI: 0.23-0.58), DOR 6 (95% CI: 3-13), and 0.78 (95% CI: 0.74-0.81) (**Table 2B**).

**Table 2A:** Subgroup analysis of the diagnostic accuracy of lncRNAs in IBD.

Subgroup	No of studies	Sen (95% CI)	Spec (95% CI)	PLR (95% CI)	NLR (95% CI)	DOR (95% CI)	AUC (95% CI)
Country							
Egypt	8	0.95 (0.86, 0.98)	0.98 (0.90, 1.00)	55.0 (9.0, 335)	0.05 (0.02, 0.16)	102 (72, 1458)	0.99 (0.98, 1.00)
Others	3	–	–	–	–	–	–
Specimen							
Serum	8	0.95 (0.86, 0.98)	0.98 (0.90, 1.00)	55.0 (9.0, 335)	0.05 (0.02, 0.16)	102 (72, 1458)	0.99 (0.98, 1.00)
Others	3	–	–	–	–	–	–
lncRNA type							
lncRNA H19	5	0.91 (0.86, 0.95)	0.97 (0.89, 0.99)	28.1 (7.9, 100.3)	0.09 (0.06, 0.14)	314 (75, 1309)	0.93 (0.90, 0.95)
Others	6	0.98 (0.77, 1.00)	1.00 (0.29, 1.00)	291 (0.4, 2079)	0.02 (0.01, 0.28)	132 (4, 439)	1.00 (0.99, 1.00)
Sample size							
≤100	7	0.91 (0.87, 0.94)	0.98 (0.90, 0.99)	36.6 (8.4, 159)	0.09 (0.06, 0.14)	411 (84, 2019)	0.93 (0.90, 0.95)
>100	4	0.99 (0.46, 1.00)	0.99 (0.33, 1.00)	104.1 (0.5, 219)	0.01 (0.01, 1.13)	989 (1, 7661)	1.00 (0.99, 1.00)
Cut-off value							
Reported	6	0.96 (0.80, 0.99)	0.98 (0.85, 1.00)	45.0 (5.5, 370)	0.04 (0.01, 0.23)	119 (31, 461)	0.99 (0.98, 1.00)
Not reported	5	0.91 (0.84, 0.95)	0.99 (0.71, 1.00)	75.9 (2.3, 2557)	0.09 (0.05, 0.17)	800 (18, 3536)	0.94 (0.92, 0.96)
Participants							
UC	7	0.92 (0.83, 0.96)	0.98 (0.77, 1.00)	47.2 (3.2, 689)	0.08 (0.04, 0.18)	585 (24, 1420)	0.97 (0.96, 0.98)
CD	4	0.97 (0.76, 1.00)	0.99 (0.70, 1.00)	123.8 (2.8, 857)	0.03 (0.01, 0.30)	476 (15, 1548)	1.00 (0.99, 1.00)

**Table 2B:** Subgroup analysis of the diagnostic accuracy of circRNAs in IBD.

Subgroup	No of studies	Sen (95% CI)	Spec (95% CI)	PLR (95% CI)	NLR (95% CI)	DOR (95% CI)	AUC (95% CI)
Specimen							
PBMCs	8	0.65 (0.59, 0.70)	0.73 (0.63, 0.81)	2.4 (1.8, 3.2)	0.48 (0.42, 0.55)	5 (3, 7)	0.72 (0.68, 0.76)
Others	2	–	–	–	–	–	–
Sample size							
≤100	6	0.64 (0.59, 0.69)	0.75 (0.67, 0.82)	2.6 (1.9, 3.6)	0.48 (0.41, 0.56)	5 (4, 9)	0.70 (0.66, 0.74)
>100	4	0.75 (0.61, 0.85)	0.68 (0.53, 0.80)	2.3 (1.6, 3.4)	0.37 (0.23, 0.58)	6 (3, 13)	0.78 (0.74, 0.81)
Cut-off value							
Reported	6	0.64 (0.59, 0.69)	0.75 (0.67, 0.82)	2.6 (1.9, 3.6)	0.48 (0.41, 0.56)	5 (4, 9)	0.70 (0.66, 0.74)
Not reported	4	0.75 (0.61, 0.85)	0.68 (0.53, 0.80)	2.3 (1.6, 3.4)	0.37 (0.23, 0.58)	6 (3, 13)	0.78 (0.74, 0.81)
Participants							
CD	8	0.66 (0.59, 0.73)	0.77 (0.71, 0.82)	2.9 (2.3, 3.6)	0.44 (0.36, 0.54)	6 (4, 10)	0.79 (0.75, 0.82)
UC	2	–	–	–	–	–	–

**Figure 7:**
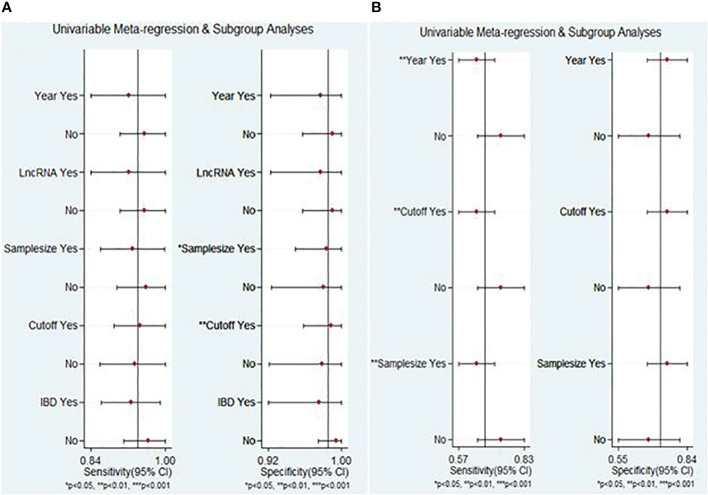
Meta-regression analysis for sensitivity and specificity of lncRNAs **(A)** and circRNAs **(B)**.

The meta-regression analysis results of lncRNA markers indicated that factors contributing to heterogeneity for specificity encompassed sample size and cut-off value determination (P < 0.05) (**Figure 7A**). Conversely, the publication year, cut-off value determination, and sample size were identified as the sources of heterogeneity of circRNA markers, particularly for sensitivity (P < 0.05) (**Figure 7B**).

### Sensitivity analysis and publication bias

**Figure 8:**
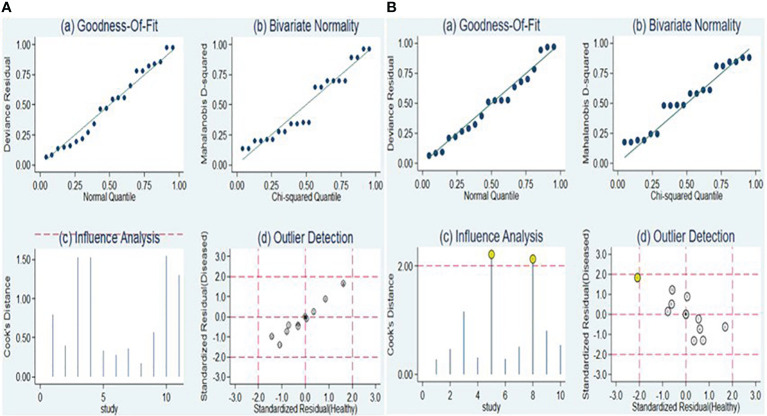
Sensitivity analysis of included studies for lncRNAs **(A)** and circRNAs **(B)**.

The sensitivity analysis is illustrated in **Figures 8A**, **B**. Examination of goodness-of-fit and bivariate normality indicated the strength and reliability of the bivariate mixed-effects model for conducting meta-analysis [**Figure 8A** (a and b), **Figure 8B** (a and b)]. Outlier identification for lncRNA markers revealed no outliers were seen indicting the findings were reliable [**Figure 8A** (d)]. On the other hand, the identification of outliers in the case of circRNA pointed to potential outlier in the form of a study conducted by Ye et al. (circRNA-103765) [**Figure 8B** (d)]. Upon removing this outlier, we observed no substantial alterations in the overall sensitivity (0.66, 95% CI: 0.60-0.72), specificity (0.75, 95% CI: 0.69-0.80), PLR (2.6, 95% CI: 2.1-3.4), NLR (0.45, 95% CI: 0.38-0.54), DOR (6, 95% CI: 4-8), and AUC (0.76, 95% CI: 0.72-0.80). This suggests that the sensitivity of the studies included was consistently low, and the results became more resilient and trustworthy.

**Figure 9:**
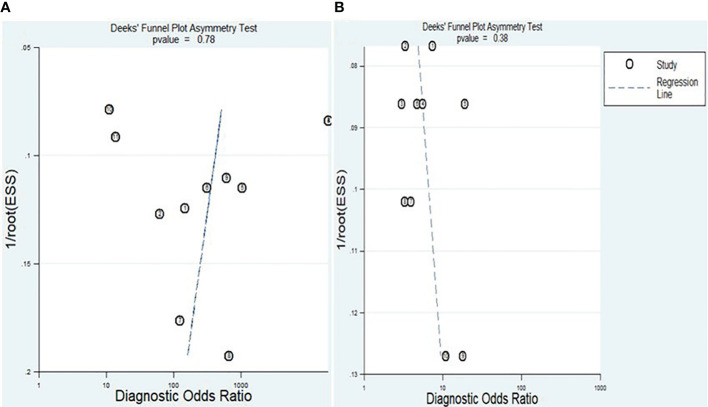
Deek’s funnel plot of publication bias analysis for lncRNAs **(A)** and circRNAs **(B)**.

The Deeks’ funnel plot asymmetry test was conducted to assess the presence of publication bias. The obtained P-values of 0.78 and 0.38 for lncRNAs and circRNA, respectively, suggest that there was no apparent indication of publication bias within the included studies (**Figures 9A, B**).

## Discussion

The burden of IBD is raising globally (47). Achieving an early diagnosis of IBD presents a formidable challenge, primarily due to the frequent misinterpretation of its symptoms as indicative of common infections (48, 49). Moreover, limited availability of diagnostic modalities may further delay prompt identification of IBD cases (50, 51). Diagnosis of IBD predominantly depends on clinical evaluation, blood tests (such as complete blood count, c-reactive protein, and erythrocyte sedimentation rate), endoscopic procedures (colonoscopy, sigmoidoscopy, and upper endoscopy), radiological imaging (X-rays, computed tomography (CT) scans), and biopsies (52–54). However, these techniques have several limitation and challenges including the invasiveness of procedures, the potential for false-negative or false-positive results, and the inability to consistently distinguish between CD and UC, necessitating a multifaceted approach for accurate diagnosis and classification (55). Thus, to embark a more accurate and timely detection of IBD, the investigation of novel diagnostic biomarkers has recently become vital, enabling early intervention and improved patient outcomes. In this regard, lncRNAs and circRNAs have gained substantial attention attributable to their diverse roles in the regulation of gene expression and pathophysiology of inflammatory processes (56, 57). LncRNAs and circRNAs with dysregulated expression play a role in diverse complex diseases, including cancer (58–62), neurodegenerative diseases (63), autoimmune diseases (64), cardiovascular diseases (65–67), inflammations and various infections (67, 68).

LncRNAs and circRNAs exhibit typical features that render them promising diagnostic biomarkers for IBD. These features include the noncoding nature of both lncRNAs and circRNAs that allow them to actively involve in gene expression regulation (69, 70), and stability in various body fluids making them readily accessible for noninvasive detection (71, 72). Besides, these biomarkers are resistant to enzymatic degradation (73).

Both lncRNAs and circRNAs are not sufficiently investigated in the context of IBD (74). While few systematic reviews have incorporated specific lncRNAs and circRNAs that exhibit altered expression in individuals with IBD compared to controls (23, 75, 76), none of these reviews have presented information on the diagnostic efficacy of these biomarkers. Thus, this study appears to be the first meta-analysis to investigate the utility of lncRNAs and circRNAs in the diagnosis of IBD. This systematic review and meta-analysis combined findings of existing studies to comprehensively assess the diagnostic potential of lncRNAs and circRNAs for the early detection and stratification of IBD patients.

In this study, a total of 11 articles encompassing 21 studies which involved 1239 IBD patients and 985 healthy controls were investigated. The findings of this meta-analysis enrolling four distinct lncRNAs and one lncRNA panel from a total of 11 studies depicted that lncRNAs could be used as potential biomarker in diagnosing IBD, with a high level of pooled sensitivity 0.94 (95% CI: 0.87-0.97) and specificity 0.99 (95% CI: 0.89-1.00), along with PLR, NLR, DOR, and AUC values of 64.25 (95% CI: 7.39-558.66), 0.06 (95% CI: 0.03-0.13), 1055.25 (95% CI: 70.61-15770.77), and 0.99 (95% CI: 0.97-0.99), respectively. On the other hand, the overall findings of our analysis enrolling eight distinct circRNAs from 10 studies showed that the pooled sensitivity, specificity, PLR, NLR, DOR, and AUC of circRNAs in the diagnosis of IBD were 0.68 (95% CI: 0.61-0.73), 0.73 (95% CI: 0.65-0.79), 2.47 (95% CI: 1.94-3.16), 0.45 (95% CI: 0.38-0.53), 5.54 (95% CI: 3.88-7.93), and 0.75 (95% CI: 0.71-0.79), respectively. The findings revealed that circRNAs illustrate a moderate level of pooled sensitivity and specificity in serving as a diagnostic marker of IBD.

The PLR value of 64.25 suggests that the likelihood of detecting positive lncRNAs in patients with IBD is around 64 times higher compared to healthy controls. Conversely, the NLR, at 0.06, implies that cases with negative test results have an approximately 6% chance of developing IBD. The DOR serves as an indicator of the discriminatory performance of a test (77), and a DOR value exceeding 1 signifies a more effective diagnostic test. In this context, the DOR of 1055.25 underscores the extreme capability of lncRNAs to effectively and efficiently discriminate between patients with IBD and their healthy counterparts. Furthermore, the AUC value serves as a reliable indicator within the assessment system. An ideal test, exhibiting perfect and flawless discrimination, would achieve an AUC of 1.0 (78). As the AUC value of a test approaches 1.0, there is a corresponding increase in the overall efficacy of the test (79). In this study, it was observed that lncRNA holds potential for differentiating IBD patients from healthy controls, as evidenced by a huge AUC value of 0.99. This proximity to 1.0 implies a substantial capacity of lncRNAs to discern effectively between individuals with IBD and those without the condition, signifying lncRNAs as an optimal test.

On the other hand, in the case of circRNAs, the probability of positive circRNA determination in IBD patients is approximately 2.47 times higher (PLR values of 2.47) compared to healthy controls, with NLR of 0.45 denoting that cases partaking negative test results have around 45% chance of developing IBD. The DOR index of 5.54 showed the relative ability of circRNAs to discriminate between patients with IBD and healthy controls. Additionally, an AUC value of 0.75 is indicative of a relative ability of cirRNAs to distinguish IBD patients from health controls. However, such findings of circRNAs and the interpretations drawn from circRNA-related investigations in this meta-analysis may not be conclusive as the number of included studies is limited. To establish more robust conclusions, it is advisable to validate these findings through a large number of studies.

In the subgroup analysis, lncRNA H9 showed a relatively high diagnostic performance (AUC: 0.93, 95% CI: 0.90-0.95) for IBD compared to other lncRNAs and lncRNA clusters. This may be due to the specific expression patterns and biological roles of lncRNA H9 in the context of IBD. However, due to the limited number of studies reporting performances of lncRNA panel and individual lncRNAs including lncRNA THRIL, lncRNA ANRIL and lncRNA PVT1, we could not able to assess their combined diagnostic performances. Consequently, our current evidence showed that lncRNA H9 may be a better and more suitable diagnostic marker for IBD.

Our finding also revealed a substantial difference in diagnostic ability of lncRNAs between the studies that had sample size >100 participants and ≤100 participants. Accordingly, studies with sample size >100 (AUC: 1.00 (95% CI: 0.99-1.00) are found to have more diagnostic performance seamlessly becoming very close to the ideal test compared with studies with sample size ≤100 (AUC: 0.93, 95% CI: 0.90-0.95). Similarly, our study showed a significant difference in diagnostic ability of circRNA between the groups with a sample size > 100 (AUC: 0.78) and ≤ 100 (AUC: 0.70). The possible justification for such discrepancy could be differences in statistical power and sampling bias that arise as a result of variances in sample size. However, large-scale studies deploying large sample size will be paramount to confirm and validate these findings.

In fact, both PLR and AUC are vital diagnostic measures with significant clinical implications. Clinically, a higher PLR enhances the utility of a diagnostic test by indicating that a positive result strongly supports the presence of the condition. It aids in patient stratification, contributing to more accurate identification of individuals who truly have the disease. A higher AUC, a key metric in ROC analysis, signifies the test’s superior ability to differentiate between individuals with and without the condition. Clinicians use the AUC to assess diagnostic accuracy, guide decisions on test suitability for patient care, and determine optimal cutoff values for clinical application (78). However, there are potential sources of bias and limitations in calculating PLR and AUC measures, including study population, verification, choice of different threshold values for defining a positive test result, variations in disease prevalence in different populations, reference standard related bias, publication bias, inclusion of small sample size, and heterogeneity in study designs, patient populations, or test methodologies (80).

Additionally, lncRNAs demonstrated notable efficacy in the diagnosis of both UC and CD with minimal differences in parameters and high AUC values (AUC: 0.97 for UC and AUC: 1.00 for CD). This indicates the possibility of using lncRNA biomarkers for all forms of IBD and their ability to diagnose both UC and CD with high sensitivity and specificity. Such findings are indicative of the applicability of lncRNAs to diagnose IBD and their possible wide spread use.

Facilitating clinical decision-making stands as the paramount value of biomarkers. Likelihood ratios offer valuable insights to clinicians, furnishing information on the probability that a patient with a positive or negative test truly has or does not have IBD (81). In evaluating the clinical utility of lncRNAs for diagnosing IBD, this study condensed and assessed PLR and NLR. A PLR > 10 and NLR < 0.1 signify a high level of diagnostic accuracy. Notably, this study showed that lncRNA H19 exhibited high diagnostic accuracy and clinical applicability. Consequently, lncRNA H19 emerges as a promising candidate deserving of further research in the realm of lncRNAs.

In this meta-analysis, the Fagan’s nomogram for lncRNAs, assuming a pre-test probability set at 20%, yielded post-test probabilities with PLR and NLR values of 0.94 and 0.01, respectively. This finding illustrates potential outcomes wherein samples testing positive for the presence of lncRNAs indicate a 94% probability of IBD development in patients, while the post-test probability of the disease decreases to 1% when samples tested negative for lncRNAs. Accordingly, lncRNAs exhibit a discernible diagnostic potential in distinguishing patients with IBD from healthy controls, and can be used as a viable screening method for IBD.

This meta-analysis revealed that lncRNAs exhibit enormously higher diagnostic performance, characterized by markedly higher sensitivity and specificity compared to circRNAs, which demonstrated moderate sensitivity and specificity values. Investigations of dysregulated expressions of lncRNAs and circRNAs are vital tools in enabling the early diagnosis of IBD and improving overall health outcomes, establishing them as promising diagnostic markers. These biomarkers are beneficial than the conventional methods, which are often invasive and exhibit lower diagnostic efficacy. This advantage is mainly attributed to the easy accessibility of peripheral blood, tissue samples, and body fluids, the consistent expression of biomarkers in these samples (82), their tissue-specific characteristics, and reliability of the qRT-PCR technique in detecting circulating biomarkers (26).

This study has several limitations. Firstly, limited number of eligible studies were used for assessing the diagnostic performance of circRNA which could have constrained and hindered the evaluation. Secondly, the data extracted from the eligible studies were relatively limited due to variations in the cutoff values of the biomarkers, which could possibly lead to potential heterogeneity. Thirdly, the eligible studies originated from limited number of countries, potentially compromising the appropriateness and generalizability of the biomarkers’ diagnostic performance to IBD patients globally. Fourthly, the statistical power of our meta-analysis could be constrained as a result of the small sample size pertained by most of the included studies. Fifthly, the unavailability of a substantial number of similar lncRNA and circRNA biomarkers prevented the pooling of results, making it challenging to identify specific single biomarkers or panels as the optimal diagnostic tools for IBD. Lastly, potential biases in the included studies related with selection, verification, reference standard usage, inappropriate index test dependency, variable diseases prevalence or characteristics, and publication related bias may impact our overall analysis. While these limitations may have impacted our meta-analysis findings, we anticipate that this study will offer baseline data for forthcoming studies. As a result, our results should be interpreted with caution, and we recommend future researchers to confirm and validate our findings through more extensive studies with larger sample sizes, following a more standardized approach.

In conclusion, our study evidenced that lncRNAs have high diagnostic accuracy in distinguishing patients with IBD from healthy controls, while circRNAs showed moderate diagnostic accuracy. This suggests that lncRNAs could serve as effective non-invasive biomarkers for IBD. Notably, both lncRNA H19 and lncRNAs in studies with a large sample size (>100) demonstrate higher diagnostic potential in the diagnosis of IBD. Additionally, lncRNAs displayed notable efficacy in the diagnosis of both UC and CD, with minimal differences in parameters. However, to validate our findings and confirm the clinical utility of lncRNAs and circRNAs in IBD diagnosis, a comprehensive array of prospective, multi-center studies, encompassing both individual and combined lncRNA and circRNA assays, should be undertaken. Furthermore, to mitigate these limitations, future research endeavors should focus on broadening the pool of eligible studies, ensuring diverse global representation, minimizing potential biases, standardizing cutoff values, and incorporating larger sample sizes. Additionally, exploration of a broader array of lncRNAs and circRNAs biomarkers from noninvasive specimens while ensuring consistent methodologies, and promoting transparent reporting practices are imperative to enhance the robustness and generalizability of future research outcomes and facilitate the translation of these biomarkers into clinically meaningful diagnostic tools for IBD.

## Data availability statement

The original contributions presented in the study are included in the article/**Supplementary Material**. Further inquiries can be directed to the corresponding author.

## Author contributions

MB: Conceptualization, Data curation, Formal analysis, Funding acquisition, Investigation, Methodology, Project administration, Resources, Software, Supervision, Validation, Visualization, Writing – original draft, Writing – review & editing. ST: Data curation, Formal analysis, Methodology, Software, Supervision, Writing – review & editing. MT: Data curation, Formal analysis, Investigation, Software, Validation, Writing – review & editing. AG: Data curation, Formal analysis, Investigation, Methodology, Software, Validation, Writing – review & editing. AS: Data curation, Formal analysis, Investigation, Software, Supervision, Visualization, Writing – review & editing. ZM: Data curation, Formal analysis, Methodology, Software, Validation, Visualization, Writing – review & editing. MW: Formal analysis, Methodology, Software, Supervision, Validation, Visualization, Writing – review & editing. SG: Data curation, Formal analysis, Investigation, Methodology, Resources, Software, Writing – review & editing. HD: Formal analysis, Investigation, Methodology, Resources, Software, Writing – review & editing. MA: Data curation, Formal analysis, Software, Validation, Visualization, Writing – review & editing. EA: Conceptualization, Data curation, Formal analysis, Funding acquisition, Investigation, Methodology, Project administration, Resources, Software, Supervision, Validation, Visualization, Writing – original draft, Writing – review & editing.
