# Supplementary material for: Long noncoding RNAs and circular RNAs as potential diagnostic biomarkers of inflammatory bowel diseases: a systematic review and meta-analysis
Source: Front Immunol. 2024 Mar 8;15:1362437. doi: 10.3389/fimmu.2024.1362437 (PMC10957631; doi:10.3389/fimmu.2024.1362437)
Supplement: Supplementary file 2 [file Table_2.docx]

**Table a: Search strategy used to retrieve eligible studies.**

| **Electronic database** | **Combination** | **Number of articles** | **Last searching date** |
| --- | --- | --- | --- |
| PubMed | (("long noncoding RNA" OR "long non-coding RNA" OR "long non coding RNA" OR "long ncRNA" OR "long ncRNAs" OR "ncRNAs, long" OR "lnc RNA" OR "RNA, long noncoding" OR "noncoding RNA, long" OR lncRNA OR ncRNA OR "circular RNA" OR "circular RNAs" OR "RNA, circular" OR circRNA OR circRNAs)) AND (("diagnos*")) AND (("inflammatory bowel disease*" OR "ulcerative colitis" OR crohn*)) | 249 | November 05, 2023 |
| EMBASE | ('chikungunya virus infection':ti,ab,kw OR 'chikungunya fever':ti,ab,kw OR 'chikungunya virus':ti,ab,kw OR 'chikungunya infection':ti,ab,kw OR 'arboviruses':ti,ab,kw OR 'CHIKV':ti,ab,kw) AND ('horn of Africa':ti,ab,kw OR 'Ethiopa':ti,ab,kw OR 'Eritrea':ti,ab,kw OR 'Djibouti':ti,ab,kw OR 'Uganda':ti,ab,kw OR 'Somalia':ti,ab,kw OR 'Sudan':ti,ab,kw OR 'South Sudan':ti,ab,kw OR 'Kenya':ti,ab,kw) | 65 | November 05, 2023 |
| Scopus | (TITLE-ABS-KEY ("long noncoding RNA" ) OR TITLE-ABS-KEY ( "long non-coding RNA" ) OR TITLE-ABS-KEY ( "long non coding RNA" ) OR TITLE-ABS-KEY ( "long ncRNA" ) OR TITLE-ABS-KEY ( "long ncRNAs" ) OR TITLE-ABS-KEY ( "ncRNAs, long" ) OR TITLE-ABS-KEY ( "lncRNA" ) OR TITLE-ABS-KEY ( "lnc RNA" ) OR TITLE-ABS-KEY ( "ncRNA" ) OR TITLE-ABS-KEY ( "RNA, long noncoding" ) OR TITLE-ABS-KEY ( "noncoding RNA, long" ) OR TITLE-ABS-KEY ( "circular RNA" ) OR TITLE-ABS-KEY ( "circular RNAs" ) OR TITLE-ABS-KEY ( "RNA, circular" ) OR TITLE-ABS-KEY ( "circRNA" ) OR TITLE-ABS-KEY ( "circRNAs" ) AND TITLE-ABS-KEY ( "diagnos*" ) AND TITLE-ABS-KEY ( "inflammatory bowel disease*" ) OR TITLE-ABS-KEY ( "ulcerative colitis" ) OR TITLE-ABS-KEY ( "crohn*" )) | 73 | November 05, 2023 |
| Other sources | “long noncoding RNA” and “diagnosis” and “inflammatory bowel disease”; “long noncoding RNA” and “diagnosis” and “ulcerative colitis”; “long noncoding RNA” and “diagnosis” and “crohn’s disease”; “circular RNA” and “diagnosis” and “inflammatory bowel disease”; “circular RNA” and “diagnosis” and “ulcerative colitis”; “circular RNA” and “diagnosis” and “crohn’s disease” | 47 | November 05, 2023 |
